# Genomic Characteristics and Comparative Genomics Analysis of *Parafenestella ontariensis* sp. nov.

**DOI:** 10.3390/jof8070732

**Published:** 2022-07-14

**Authors:** Evgeny Ilyukhin, Svetlana Markovskaja, Abdallah M. Elgorban, Salim S. Al-Rejaie, Sajeewa S.N. Maharachchikumbura

**Affiliations:** 1 Department of Biological Sciences, Brock University, St. Catharines, ON L2S 3A1, Canada; 2 Laboratory of Mycology, Nature Research Centre, LT 08406 Vilnius, Lithuania; svetlana.markovskaja@gamtc.lt; 3 Department of Botany and Microbiology, College of Science, King Saud University, Riyadh 11451, Saudi Arabia; aelgorban@ksu.edu.sa; 4 Department of Pharmacology and Toxicology, College of Pharmacy, King Saud University, Riyadh 11451, Saudi Arabia; rajaie@ksu.edu.sa; 5 Center for Informational Biology, School of Life Science and Technology, University of Electronic Science and Technology of China, Chengdu 611731, China; sajeewa@uestc.edu.cn

**Keywords:** comparative genomics, *Cucurbitariaceae*, Dothideomycetes, fungal lifestyle

## Abstract

A new ascomycetous species of *Parafenestella* was isolated from *Acer negundo* during the survey of diseased trees in Southern Ontario, Canada. The species is morphologically similar to other taxa of *Cucurbitariacea* (*Pleosporales*). The new species is different from the extant species in the morphology of ascospores, culture characteristics and molecular data. The novel species is described as *Parafenestella ontariensis* sp. nov. based on morphological and multi-gene phylogenetic analyses using a combined set of ITS, LSU, *tef1* and *tub2* loci. Additionally, the genome of *P. ontariensis* was sequenced and analyzed. The phylogenomic analysis confirmed the close relationship of the species to the fenestelloid clades of *Cucurbitariaceae*. The comparative genomics analysis revealed that the species lifestyle appears to be multitrophic (necrotrophic or hemi-biotrophic) with a capability to turn pathogenic on a corresponding plant host.

## 1. Introduction

The genus *Parafenestella* typified by *P. pseudoplatani* was introduced by Jaklitsch et al. [1]. The genus is included in the family of *Cucurbitariaceae* (*Pleosporales*) which is characterized by ostiolate ascomata seated on basal stromatic structure, with cylindrical asci and pigmented, muriform ascospores [2]. The recent studies discovered a number of new species of *Parafenestella* on different plant hosts worldwide [3,4]. There are 15 species epithets in Index Fungorum, but only 14 species of *Parafenestella* are legitimate. *Parafenestella mackenziei* was synonymized with *P. faberi* (www.indexfungorum.org, accessed 23 January 2022). The species of *Parafenestella* are mainly fungicolous (associated with perithecial ascomycetes), saprobic or necrotrophic on woody plants [1,4].

The species recognition within the genus *Parafenestella* and other *Cucurbitariaceae* is mainly based on morphology characteristics accompanied with multi-gene (SSU-ITS-LSU, *rpb2*, *tef1*, *tub2*) phylogenetic analyses. This approach reveals both cryptic and new species.

The rapidly developing new generation sequencing technologies provide high-quality, cost-effective genomic data that can be applied to different analyses. It has been found that phylogenomic approach can improve the resolution of phylogenetic trees used to resolve taxonomic uncertainties or support species reclassification [5]. One such study found out that a polyphagous plant pathogen, *Corynespora olivacea,* was previously misclassified at the family level [6]. Genomic data are also employed for comparative genomics analysis that may reveal genomic underpins for lifestyle adaptations within the set of close organisms and ultimately help define a species lifestyle [7].

The abilities to successfully colonize plant host tissues and further degrade complex plant carbohydrates are important aspects of fungal lifestyles [8]. In particular, plant-associated fungi require specific sets of genes involved in the production of various secondary metabolites (SM) and enzymes needed for interaction with a host. The genes involved in SM biosynthesis pathways are often clustered in a fungal genome [9]. SMs are bioactive, small molecules that are not essential for organism growth. However, they are involved in producing toxic compounds used by necrotrophic, polyphagous fungi to kill a range of cells in plant hosts [10]. Identifying the pathways involved in the production of SMs via the search for the genes encoding key enzymes (e.g., polyketide synthase (PKS)) is crucial for understanding fungus’ capability to swiftly infect a plant host. The SM complement in fungi was found to be specific to different lifestyles [11].

Carbohydrate-active enzymes (CAZYs) play an important role in the invasion of plant hosts by fungi. Fungal species can produce different kinds of CAZYs involved in the breakdown, biosynthesis or modification of glycoconjugates, oligo- and polysaccharides [12]. Plant pathogenic and endophytic fungi produce various enzymes (e.g., glycoside hydrolase (GH)) to deconstruct cell wall polysaccharide families involved in cellulose and hemicellulose hydrolysis. CAZY content also varies among fungal species and often shows adaptation to nutrition type [13].

It is assumed that a comprehensive comparison of the abundance and distribution of SMs and CAZYs between the *Cucurbitariaceae* species and the model species with different lifestyles will reveal the *Parafenestella ontariensis*’ lifestyle and its aptitude to be pathogenic on a related plant host. To date, no draft genome sequences have been published for *Parafenestella* spp. The availability of a high-quality draft genome sequence of *P. ontariensis* alongside the genomes of other species of *Cucurbitariaceae* will also contribute to the understanding additional aspects of plant–fungus interactions (e.g., virulence determinants) for this little-studied family.

This paper describes a new species of *Parafenestella* collected from *Acer negundo* in Ontario, Canada. Both morphology characteristics and phylogenetic analysis of four-gene sequence data support the introduction of the new species, *P. ontariensis*. The new taxon was established based on the recent recommendations for description of novel fungal species [14]. The phylogenomic analysis using the protein sequences of *P. ontariensis* confirmed the species position in *Cucurbitariaceae* (*Pleosporales*) and its close relationship to the fenestelloid taxa of the family. For comparative genomics analysis, *P. ontariensis* was included in a subset of the *Cucurbitariaceae* species (*Cucurbitaria berberidis* and *Fenestella fenestrata)* for better data representation. The analysis revealed that the lifestyle of the members of the *Cucurbitariaceae* family could be both necrotrophic and hemi-biotrophic (multitrophic), with a capability to become pathogenic on a corresponding plant host.

## 2. Materials and Methods

### 2.1. Sample Collection and Isolation

During the survey of forestry areas in the Niagara region (Ontario, Canada) in April 2020, the branches of *A. negundo* expressing dieback symptoms were collected for further study. Single spore isolates from the sampled ascomata were obtained following the method described in Phookamsak et al. [15]. The isolates were plated on malt extract agar (MEA) and incubated at room temperature (20–25 °C) in the dark. Germinating ascospores were aseptically transferred onto fresh MEA plates and stored under the same conditions. The purified representative isolate EI-6 was selected for the species description, studying micro-morphology of asexual morph and sequencing. The specimen was deposited in the Herbarium of the Nature Research Centre, Institute of Botany, Vilnius, Lithuania and the Canadian National Mycological Herbarium, Ottawa, ON, Canada.

### 2.2. Morphological Analysis

The morphological description was performed using microscopy techniques with dissecting (AmScope SE306R-PZ) and compound (AmScope B120C-E5) microscopes. Microscopic observations were made in distilled water. The description and measures of asexual stage were carried out using the pure cultures of the representative isolate. Fruiting structures (10 ascomata with ostioles and 10 pycnidia) were measured for the macromorphological characteristics. Micromorphological structures of sexual (20 asci, 50 ascospores) and asexual (50 conidia) morph were further measured. The obtained measures were recorded as a range of minimum and maximum values in parentheses. Picturing was performed with a 5 MP digital AmScope camera MD500 supplied with AmScopeX software (AmScope, Irvine, CA, USA).

### 2.3. DNA Extraction, PCR Amplification and Sequencing

Genomic DNA (gDNA) was extracted from pure culture using DNeasy PowerSoil Pro Kit (Qiagen, Hilden, Germany) following the manufacturer’s instructions. The internal transcribed spacer (ITS) region was amplified with the primer pair ITS1/ITS4 [16]. The primer pair LR0R/LR5 [17] was used to amplify large subunit rRNA (LSU). PCR amplifications were executed using C-1000 thermal cycler (Bio-Rad Laboratories, Hercules, CA, USA) under the conditions described in the references for each region. The quality of PCR products was examined using electrophoresis in 1% agarose gel. All the procedures and Sanger sequencing were carried out at Genome Quebec Innovation Centre (Montreal, QC, Canada).

### 2.4. Sequence Alignment and Phylogenetic Analysis

The isolate identification was first conducted by standard BLASTn search using the obtained ITS and LSU sequences. Then, the sequences of all available *Parafenestella* spp. were extracted to perform phylogenetic analysis. The sequence dataset of the fenestelloid species [4] was further employed to build the final sequence matrix. The sequences were initially aligned using CLUSTAL-X2 [18] and manually edited employing MEGA-X [19]. Some characters were trimmed from both ends of the alignments to approximate the length of the obtained sequences to those included in the dataset. Phylogenetic analyses were executed using randomized accelerated maximum likelihood (RAxML) v. 8.0 method [20] for maximum likelihood (ML) analysis, and MEGA-X [19] was employed for maximum parsimony (MP) analysis. Bayesian posterior (BP) probabilities were defined using MrBayes v.3.2.7 [21].

The ML analysis was performed using the transitional unequal (TIM2) substitution model [22] with gamma-distributed rate of heterogeneity and proportion of invariant sites selected with ModelTest-NG v.0.1.7 [23]. The branch support was estimated with bootstrapping of 1000 replicates [24]. To execute the MP analysis, heuristic search with random sequence additions and tree bisection and reconnection algorithm were selected. Max-trees were set to 500, branches of zero length were collapsed and all equally parsimonious trees were saved. The BP values were defined by Markov chain Monte Carlo (MCMC) sampling with the general time reversible (GTR) model. Six simultaneous Markov chains were run for 100,000 generations. The first 1000 trees were discarded and the remaining trees were used to calculate BP in the majority rule consensus tree. The phylograms were visualized using FigTree v. 1.4.4 [25]. The newly generated sequences were deposited in GenBank (Table 1). The alignments used in the analyses were submitted to TreeBase (www.treebase.org; ID: S29577; accessed 14 April 2022).

### 2.5. DNA Extraction, Library Preparation, Genome Sequencing and Assembly

Highly purified total gDNA was extracted from the fungal mycelia obtained from the 12 d-old culture of the representative isolate EI-6 using DNeasy PowerSoil Pro Kit (Qiagen, Hilden, Germany). gDNA was quantified using the Quant-iT PicoGreen dsDNA Assay Kit (Life Technologies, Carlsbad, CA, USA). Libraries were generated using the NEBNext Ultra II DNA Library Preparation Kit for Illumina (New England Biolabs, Ipswich, MA, USA). The genome was sequenced with the Illumina NovaSeq 6000 platform using 150 bp pair-end sequencing strategy. All the procedures were performed at Genome Quebec Innovation Centre (Montreal, Canada). The raw reads were filtered with Trimmomatic v. 0.38.1 [26] and assembled into scaffolds using SPAdes v. 3.12.1 [27]. The genome assembly characteristics were accessed using QUAST v. 5.0.2 [28]. Repeat sequence content was identified with RepeatMasker v. 4.0.9 [29] using the Repbase library of repeats for fungi [30]. The completeness of assembly was estimated with BUSCO v.4.0.5 based on the dataset ascomycota_odb10 [31].

### 2.6. Gene Prediction and Functional Annotation

Gene prediction was performed using Augustus v. 3.4.0 [32]. Augustus was trained with the gene structures of the representative genome of *C. berberidis* (CBS 394.84) which were obtained with the MAKER2 pipeline v. 2.31.11 [33]. The EuKariotic Orthologous Group (KOG) distribution was identified employing the eggNOG 5.0 dataset [34]. The CAZY annotation was performed using dbCAN2 with the HMMER-based classification tool [35]. The SM backbone genes were predicted using the SMIPS tool [36].

### 2.7. Phylogenomic Analysis and Genome Sequence Alignment

The recently published protein sequence dataset of *Pleosporales* species [6] with some modifications was employed for the phylogenomic analysis. The proteomes for 18 species were retrieved from the MycoCosm portal [37] and GenBank genome database (Table 2). Orthofinder v. 2.5.4 [38] was used to identify the single-copy orthogroup (SCO) within all species included in the analysis. Multiple sequence alignment was performed using MAFFT v. 7.489 [39]. The ML tree was inferred with FastTree v. 2.1.10 [40]. Phylogram was visualized employing FigTree v.1.4.4 [25]. Genome alignment between genome sequences was performed with MashMap v. 2.0 [41] and displayed as a dot-plot graph using D-Genies [42].

## 3. Results

### 3.1. Taxonomy

***Cucurbitariaceae*** G. Winter [as *Cucurbitarieae*], Rabenh. Krypt. -Fl., Edn 2 (Leipzig) 1.2: 308. 1885.

***Parafenestella*** Jaklitsch & Voglmayr, in Jaklitsch et al., Stud. Mycol. 90: 108. 2018.

**Figure 1:**
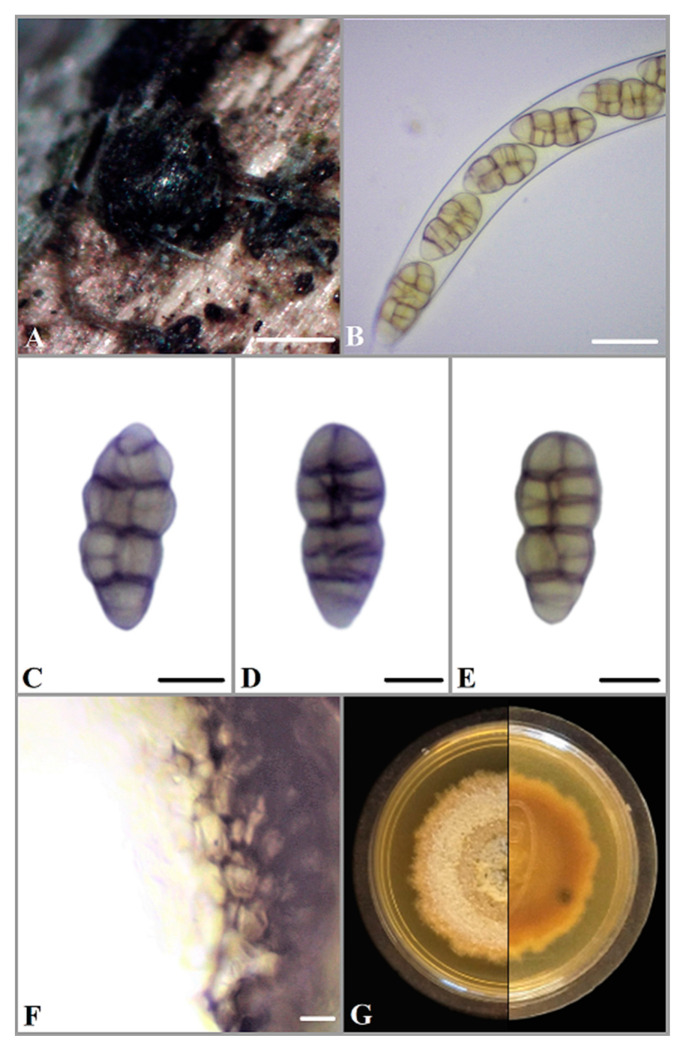
(**A**) Ascomata of *P. ontariensis* on *Acer negundo*. (**B**) Ascus. (**C**–**E**) Ascospores. (**F**) Peridium. (**G**) Fourteen-day-old culture on MEA. Scale bars: **A** = 200 μm, **B** = 20 μm, **C**–**E** = 10 μm, **F** = 5 μm.

***Parafenestella ontariensis*** Ilyukhin & Markovsk., sp. nov. Figure 1.

MycoBank number: MB 843298.

Etymology—Epithet refers to the geographical region, vicinities of the lake of Ontario (Ontario, Canada), where the species was found.

Saprophytic or pathogenic on branches of *Acer negundo*. Sexual morph: *Ascomata* (210–) 265–340(–370) μm (*n* = 10) diameter, subglobose to globose, black, solitary or aggregated in groups, immersed in bark, some on conidiomata of *Diaporthe* sp., dark brown, thick-walled and ostiolate. *Ostioles* 65–130(–150) μm (*n* = 10) diameter, indistinct, black and rounded. *Peridium* 15–45 μm thick, pseudoparenchymatous, consisting of isodiametric cells 5–10 μm wide, thick-walled, dark brown and t. angularis to globulosa. *Hamathecium* consists of numerous branched and anastomosing paraphyses, 2–3.5 μm wide with free ends. *Asci* (151–)165–195(–215) × (15.5–)17.5–20.5(–23.0) μm (*n* = 20), cylindrical, bitunicate, with an ocular chamber, short stipe and knob-like base, containing eight ascospores in uniseriate arrangement. *Ascospores* (22.5–)26.0–32.5(–36.0) × 10.0–12.0(–13.5) μm (*n* = 50), oblong, fusoid or narrowly ellipsoid with rounded ends, muriform, constricted at the median septum, usually with three to five transverse and one to two longitudinal septa, pale or yellowish brown when young, turning brown to dark brown at maturity, thick-walled and smooth.

Culture characteristics and asexual morph: colony slow-growing (13 mm) on MEA at room temperature in the dark after 7 days, initially white, dense, with thick texture, turning brownish, later grey-brown, darker at the center, covered with brown aerial mycelium; reverse side brown to grey brown. *Pycnidia* appearing after 12 days, light brown to dark brown (texture angularis), globose, solitary, rare, 65–120 in diameter (*n* = 10). *Conidia* (3.2–)3.5–4(–4.5) × (1.0–)1.2–1.5(–1.7) μm (*n* = 50), 1-celled, oblong or allantoid, sometimes attenuated towards one end, hyaline, with two subterminal drops and smooth.

Notes: Based on morphological characteristics and phylogenetic analysis, *P. ontariensis* is close to *P. alpina* sampled from the twigs of *Cotoneaster integerrimus* in Austria [4]. Both species have similar morphological characteristics but ascospores of *P. ontariensis* are slightly longer (22.5–36 µm) than those of *P. alpina* (19–35µm). *P. alpina* also has wider (10.5–15.5 µm) and more septate ascospores. Lack of brown color of the *P. alpina* cultures grown on corn malt agar (CMA) also makes these two species different [4]. Culture color of *P. alpina* is dark grey to olivaceous, meanwhile *P. ontariensis* is grey brown.

Material examined: CANADA, Ontario, St. Catharines, N43°12′45.3″, W79°14′33.6″, sexual morph (ascomata) was collected from branches of *Acer negundo*, 29 April 2020, E. Ilyukhin (**Holotype**: BILAS 51516, ex-type culture BILAS 51517 = EI-6, isotype DAOM 984987).

### 3.2. Phylogenetic and Phylogenomic Analyses

**Figure 2:**
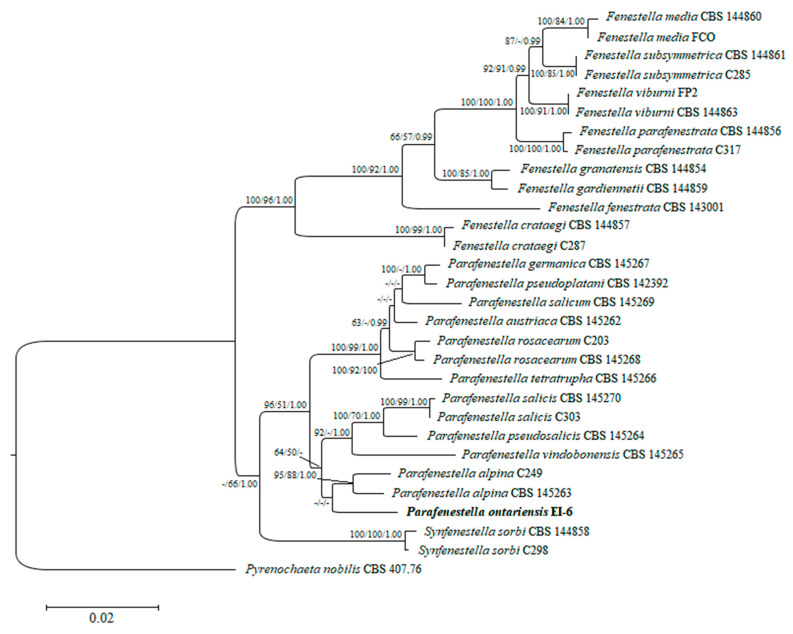
Phylogram of RAxML tree generated based on the analysis of combined ITS, LSU, *tef1* and *tub2* sequence data. Bootstrap support values for ML and MP ≥ 50% and BP ≥ 0.90 are defined as ML/MP/BP above and below the nodes. Strain of the new species is in bold. The tree is rooted to *Pyrenochaeta nobilis* (CBS 407.76).

A combined set of ITS, LSU, *tef1* and *tub2* sequence data of 29 *Cucurbitariaceae* species was analyzed to define the taxonomic placement of the isolate EI-6 and infer its relationships at the intrageneric level. The performed RAxML analysis produced the phylogenetic tree with the topology similar to the multi-gene tree obtained in the recent study on fenestelloid clades of *Cucurbitariaceae* [4]. Individually generated single gene trees did not show conflicts in phylogenetic signals as well (data not shown). The best scoring ML tree with a log likelihood value of −3046.830912 is depicted in Figure 2. The isolate of the novel species *P. ontariensis* was clustered separately and formed a subclade with *P. alpina*. The new species was nested within the well-supported clade of *Parafenestella* spp. (96% ML, 51% MP, 1.00 BP).

**Figure 3:**
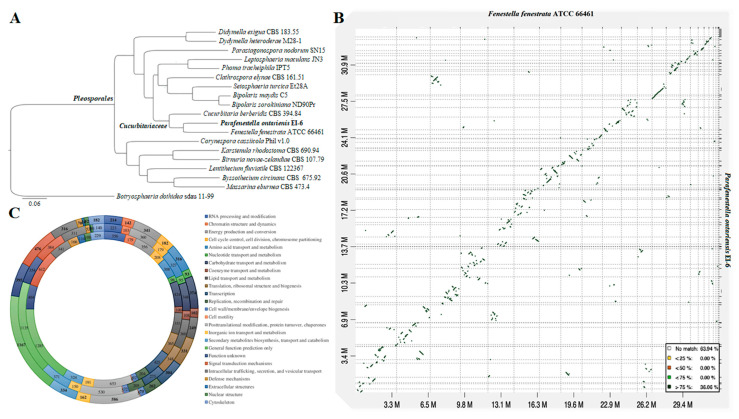
(**A**) ML tree generated from the concatenated alignment of 3 145 SCOs. (**B**) Dot-plot graph showing syntenic blocks between scaffold sequences of *F. fenestrata* and *P. ontariensis*. (**C**) Graph showing the KOG gene annotations of *C. berberidis* (inner), *F. fenestrata* (middle) and *P. ontariensis* (outer layer).

A total of 4 789 orthogroups present in all species included in the analysis with 3 145 SCOs were uncovered. The phylogenomic tree constructed from the matrix of protein sequence data has shown the strain *P. ontariensis* EI-6 clustered with *F. fenestrata* ATCC 66461. This clade was placed within the *Cucurbitariaceae* family of *Pleosporales* confirming the family level classification results obtained from the phylogenetic analysis. All the clades were formed with 100% ML support (Figure 3A).

The genome sequence comparison was performed between *P. ontariensis* and the closely allied *F. fenestrata*. The analysis showed that 36.1% of syntenic blocks were more than 75% similar with several inversions (Figure 3B).

### 3.3. Genome Sequencing and Assembly Characteristics of Parafenestella ontariensis and Other Closely Related Cucurbitariaceae Species

The draft genome sequence of *P. ontariensis* EI-6 was characterized and compared to other publicly available genomes of *Cucurbitariaceae* (*C. berberidis* CBS 394.84, *F. fenestrata* ATCC 66461). The assembly statistics of the species are presented in Table 3. The genome of *P. ontariensis* was sequenced with higher coverage (267.4×) than the genomes of *C. berberidis* and *F. fenestrata* (145.7× and 81.9×, respectively) and assembled into 162 scaffolds (>1000 bp) with the size of 34.4 Mb. That was slightly larger than the genomes of other *Cucurbitariaceae* species (32.7–32.9 Mb) arranged into 42 (*C. berberidis*) and 22 (*F. fenestrata*) scaffolds. The genome of *C. berberidis* had a lower genomic GC content (49.3%) compared to *P. ontariensis* (50.79%) and *F. fenestrata* (51.53). Despite the smaller genome size, 12, 429 gene models were predicted for *C. berberidis* and 11, 804 for *F. fenestrata*, which is a larger number than for *P. ontariensis* (11, 748). The repeat contents of the genomes were found to be relatively low (1.59% for *P. ontariensis*, 2.24% for *C. berberidis* and 3.91% for *F. fenestrata*). The genome assembly completeness assessed by BUSCO indicated that the genomes used in the analysis were nearly complete (97.2–98.1%) which makes the sequences suitable for further analysis.

Of the total protein sequences analyzed, 7 895 for *C. berberidis*, 6 473 for *F. fenestrata* and 6 694 for *P. ontariensis* were functionally assigned based on the KOG hits. The KOG analysis revealed that the protein allocation pattern of *P. ontariensis* was very similar to the other two species of *Cucurbitariaceae*, indicating that the species may have the same ecological niches and lifestyles. It has also been found that *C. berberidis* had more genes involved in carbohydrate transport and metabolism (424) as well as signal transduction (612). *F. fenestrata* had more genes related to secondary metabolite biosynthesis, transport and catabolism (371). *P. ontariensis* contained a similar number of genes related in these important processes (Figure 3C).

### 3.4. Secondary Metabolites

**Figure 4:**
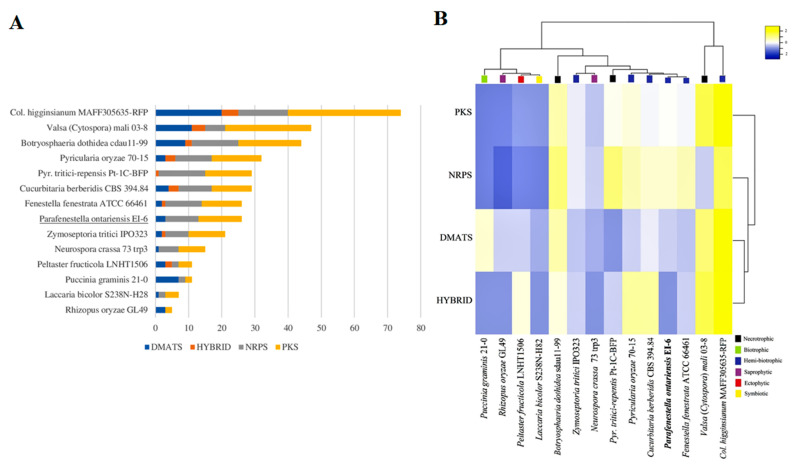
(**A**) Overall comparison of SM backbone genes in genomes of *Cucurbitariaceae* species and 11 other fungi with different lifestyles. (**B**) Heatmap graph showing SM clusters in *Cucurbitariaceae* and other fungal species with different lifestyles.

The genomes of the *Cucurbitariaceae* species, including *P. ontariensis*, were searched for the genes encoding the key enzymes (NRPS (non-ribosomal peptide synthetase), PKS (polyketidesynthase), HYBRID (PKS-NRPS) and DMATS (dimethylallyl tryptophane synthase). The identified SMs were compared to the SM content of the model species with different lifestyles (*Botryosphaeria dothidea* sdau11-99 (*B. dothidea*), *Colletotrichum higginsianum* MAff305635-RFP (*C. higginsianum*), *Laccaria bicolor* S238N-H28 (*L. bicolor*), *Neurospora crassa* 73 trp3 (*N. crassa*), *Peltaster fructicola* LNHT1506 (*P. fructicola*), *Pyrenophora tritici-repensis* Pt-1C-BFP (*P. tritici-repensis*), *Pyricularia oryzae* 70-15 (*P. oryzae*), *Puccinia graminis* 21-0 (*P. graminis*), *Rhizopus oryzae* GL49 (*R. oryzae*), *Valsa* (*Cytospora*) *mali* 03-8 (*V. mali*) and *Zymoseptoria tritici* IPO323 (*Z. tritici*)). The members of the *Cucurbitariaceae* family were found to contain a moderate number of the genes encoding SMs (29 in *C. bereberidis*, 26 in *F. fenestrata* and *P. ontariensis*). The *Cucurbitariaceae* species had a significantly lower number of SM clusters compared to the hemi-biotrophic *C. higginsianum* (74) or necrotrophic *V. mali* (47), *B. dothidea* (44). At the same time, it was notably higher than those for other lifestyles (e.g., saprotrophic *R. oryzae* (5) and symbiotic *L. laccata* (7)) (Figure 4A). Interestingly, *C. berberidis* had an almost identical complement of SMs (except DMATS) as necrotrophic *P. tritici-repensis*. The clustering analysis demonstrated that the *Cucurbitariaceae* species were grouped with hemi-biotrophic *P. oryzae*. It has also shown that the members of the *Cucurbitariaceae* family had a relatively expanded set of genes encoding NRPS (Figure 4B).

### 3.5. Carbohydrate Active Enzymes

**Figure 5:**
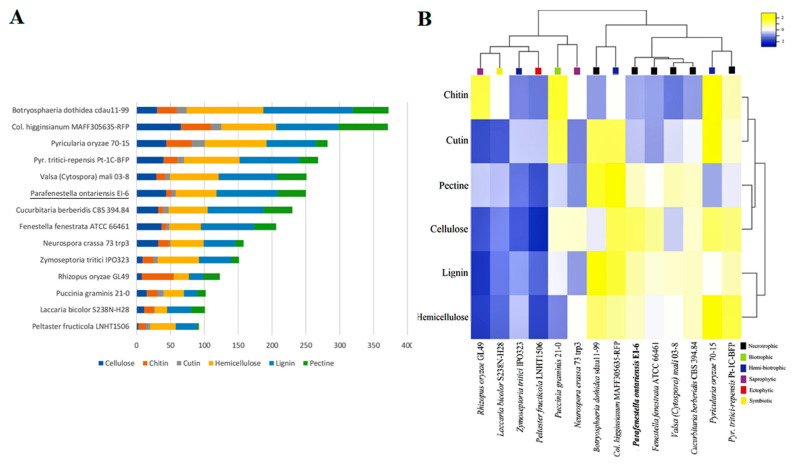
(**A**) Overall comparison of CAZY complements in genomes of *Cucurbitariaceae* species and fungi with different lifestyles. (**B**) Heatmap graph showing CAZY expansion in *Cucurbitariaceae* and 11 other fungal species related to different lifestyles.

**Figure 6:**
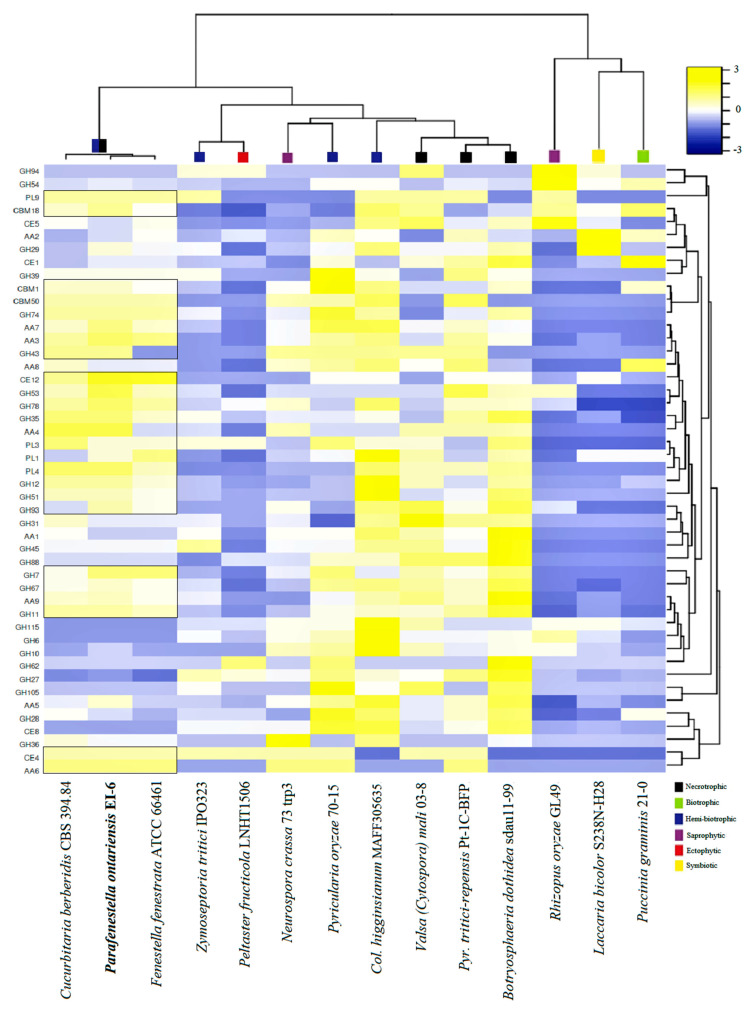
Distribution of selected plant cell wall degrading enzymes among *Cucurbitariaceae* species and other fungi with different lifestyles.

The CAZY content analysis was performed for the same set of species. It was found that the *Cucurbitariaceae* species were well equipped with the genes encoding CAZYs. The species contained an extensive set of auxiliary activity (AA) enzymes, carbohydrate esterases (CE), glycoside hydrolases (GH) and polysaccharide lyase (PL), which indicated their capacity to degrade plant cell walls effectively. In particular, the genomes of *P. ontarienis* were found to have 250 CAZY-related genes, *C. berberidis* (230) and *F. fenestrata* (206) (Figure 5A). It has also shown that the CAZY complement of *P. ontariensis* was very similar to necrotrophic *V. mali*. However, these two species carried a different numbers of genes involved in cellulose and hemicellulose degradation. Broadly, the species of *Cucurbitariaceae* had a much more expanded set of CAZYs than ectophytic *P. fructucola* (92), while it was significantly reduced compared to necrotrophic *B. dothidea* (372). The cluster analysis further confirmed the high similarity of CAZY content of the *Cucurbitariaceae* species with *V. mali* (Figure 5B). It was also demonstrated that the members of *Cucurbitariaceae* (*P. ontariensis*, especially) could encode a relatively high number of the genes involved in cellulose degradation. The further analysis of the selected CAZY families [11] has shown that the *Cucurbitariaceae* species formed a separate cluster with 25 modules (seven hemicellulose, six pectin, five cellulose, four cutin, three chitin) which are relatively expanded in at least two species in the sub-set compared to other species included in the analysis (Figure 6).

## 4. Discussion

The *Cucurbitariaceae* family is a heterogeneous group of Ascomycota (*Pleosporales*) that includes the species with necrotrophic or saprobic lifestyles on woody plants [2,43]. The recently introduced genus *Parafenestella* was characterized as a phylogenetically close taxon to *Fenestella,* mainly associated with *Diaporthales* spp [1]. Morphologically, the new species *P. ontariensis* meets the genus diagnosis of lacking well-delimited pseudostromata and ascospores that would indicate possible transition from fenestella-like to (neo) cucurbitaria-like species [4].

The genome sequence obtained for the isolate *P. ontarienis* EI-6 allowed the comparison of the species with other members of the *Cucurbitriaceae* family and the model fungal species with different lifestyles. The genome size of the *Cucurbitariaceae* species is similar to the average for Ascomycota (36.9 Mb) [44]. However, it is significantly smaller compared to some pathogenic ascomycetes (e.g., *Colletotrichum truncatum* of *Glomerellaceae* 55.4 Mb [45], *Phomopsis longicolla* of *Diaporthaceae* 64.7 Mb [46]). Considering relatively low similarity with multiple inversions between the genome sequences of *P. ontarienis* and *F. fenestrata*, a mesosynteny-like pattern can be assumed. The pattern was observed for Dothideomycetes, indicating a random reshuffling of gene content with the genes remaining conserved [47].

The fungal lifestyles can be distinguished based on the SM clusters, which were found to be notably expanded in necrotrophic and hemi-biotrophic fungal species compared to biotrophic, saprobic, ectophytic and symbiotic fungi [11]. The SMs (e.g., PKS) are involved in important processes such as producing mycotoxins needed for initial host colonization. It has also been found that PKS and NRPS are rapidly evolving genes in fungi during gene transfer, loss and duplication [48]. *P. ontariensis* and other *Cucurbitariacea* species have arsenals of SMs which are similar to both hemi-biotrophic and necrotrophic fungi. For instance, the SM content of the *Cucurbitariacea* species is close to hemi-biotrophic *P. oryzae*, a well-known fungal pathogen causing rice blast [49]. It was also observed that *C. berberidis* had the same number of SM clusters with similar distribution as *P. tritici-repensis*, a necrotrophic pathogen causing destructive foliar wheat disease [50]. Overall, the members of *Cucurbitariaceae* were well equipped with the NRPS backbone genes related to virulence processes in fungal pathogens [51].

The CAZY repertoires were also defined and classified for necrotrophic, biotrophic, hemi-biotrophic, saprophytic, ectophytic and symbiotic fungi. The fungal lifestyles were well delimited based on this classification. It has been shown that necrotrophic and hemi-biotrophic fungi have notably more genes related to plant cell wall degrading enzymes (e.g., PL1, GH61) than biotrophic and symbiotic fungi [13]. The members of the *Cucurbitariaceae* family have a relatively expanded set of CAZYs involved in penetrating plant cell walls needed for the successful colonization of a plant host. Based on the CAZY component, the *Cucurbitariaceae* species were clustered separately with the strain *V. mali* 03-8, a highly virulent pathogen causing Valsa canker on apple trees [52]. Another finding was that the *Cucurbitariaceae* species (*P. ontariensis*, especially) had a large arsenal of CAZYs involved in cellulose degradation that was previously noticed for saprotrophic fungi [53].

Based on results of the comparative genomics analysis, it can be concluded that *P. ontarienis* and other close *Cucurbitariacea* species may have a multitrophic lifestyle. A species can become necrotrophic or hemi-biotrophic under specific circumstances (e.g., host shift). This kind of lifestyle was also assumed for saprotrophic *Phomopsis liquidambari* [54] and endophytic *Sarocladium brachiariae* [55]. To confirm lifestyle switch or transition, more species of *Cucurbitariaceae* with different ecological characteristics should be sequenced and analyzed using the comparative genomics approach. The identified arsenals of SMs and CAZYs can cause the *Cucurbitariaceae* species to be pathogenic on a related plant host. However, pathogenicity assays should be performed to observe the fungus’ capability to cause a symptomatic disease that eventually leads to plant death.

## Figures and Tables

**Table 1:** Strains used in phylogenetic analysis with their GenBank numbers. Strain from this study is in bold. Holo-, neo- and epi-types are marked with *. Sequences retrieved from genome sequence (GCA_021596325) are marked with **.

Species	Strain	Host	GenBank Accession Numbers			
			ITS	LSU	*tef1*	*tub2*
*Fenestella crataegi*	CBS 144857 *	*Crataegus monogyna*	MK356282	MK356282	MK357555	MK357599
	C287	*C. monogyna*	MK356281	MK356281	MK357554	MK357598
*F. fenestrata*	CBS 143001 *	*Alnus glutinosa*	MF795765	MF795765	MF795853	MF795893
*F. gardiennetii*	CBS 144859	*Acer saccharum*	MK356283	MK356283	MK357556	MK357600
*F. granatensis*	CBS 144854 *	*Acer granatense*	MK356284	MK356284	MK357557	MK357601
*F. media*	CBS 144860 *	*Corylus avellana*	MK356285	MK356285	MK357558	MK357602
	FCO	*Carpinus orientalis*	MK356286	MK356286	MK357559	-
*F. parafenestrata*	CBS 144856 *	*Quercus robur*	MK356291	MK356291	MK357564	MK357607
	C317	*Salix* sp.	MK356292	MK356292	MK357565	MK357608
*F. subsymmetrica*	CBS 144861 *	*Acer campestre*	MK356297	MK356297	MK357569	MK357610
	C285	*Juglans regia*	MK356293	MK356293	MK357566	-
*F. viburni*	CBS 144863 *	*Viburnum lantana*	MK356300	MK356300	MK357572	MK357613
	FP2	*V. lantana*	MK356299	MK356299	MK357571	MK357612
*Parafenestella alpina*	CBS 145263 *	*Cotoneaster integerrimus*	MK356302	MK356302	MK357574	MK357615
	C249	*Salix appendiculata*	MK356303	MK356303	MK357575	MK357616
*P. austriaca*	CBS 145262 *	*Rosa canina*	MK356304	MK356304	MK357576	MK357617
*P. germanica*	CBS 145267 *	*Corylus avellana*	MK356305	MK356305	MK357577	MK357618
***P. ontariensis***	EI-6 *	***Acer negundo***	**OM286882**	**OM286884**	genome **	genome **
*P. pseudoplatani*	CBS 142392 *	*Acer pseudoplatanus*	MF795788	MF795788	MF795876	MF795914
*P. pseudosalicis*	CBS 145264 *	*Salix* cf. *alba*	MK356307	MK356307	MK357579	MK357620
*P. rosacearum*	CBS 145268 *	*Pyracantha coccinea*	MK356311	MK356311	MK357583	MK357624
	C203	*Pyrus communis*	MK356308	MK356308	MK357580	MK357621
*P. salicis*	CBS 145270 *	*Salix alba*	MK356317	MK356317	MK357589	MK357629
	C303	*S. alba*	MK356316	MK356316	MK357588	MK357628
*P. salicum*	CBS 145269 *	*S. alba*	MK356318	MK356318	MK357590	MK357630
						continued
*P. tetratrupha*	CBS 145266 *	*Alnus glutinosa*	MK356319	MK356319	MK357591	MK357631
*P. vindobonensis*	CBS 145265 *	*Salix babylonica*	MK356320	MK356320	MK357592	MK357632
*Pyrenochaeta nobilis*	CBS 407.76 *	*Laurus nobilis*	MF795792	MF795792	MF795880	MF795916
*Synfenestella sorbi*	C298	*Sorbus aucuparia*	MK356325	MK356325	MK357597	MK357636
	CBS 144858	*S. aucuparia*	MK356324	MK356324	MK357596	MK357635

**Table 2:** Strains of *Pleosporales* species used in phylogenomic analyses. Strain from this study is in bold. NA—data not available.

Species Name	Strain	GenBank Accession Number	Host	Country
*Bimuria novae-zelandiae*	CBS 107.79	GCA_010015655	NA	NA
*Bipolaris sorokiniana*	ND90Pr	GCA_000338995	NA	NA
*Bipolaris maydis*	C5	GCA_000338975	NA	NA
*Botryosphaeria dothidea*	sdau11-99	GCA_011503125	*Malus* sp.	China
*Byssothecium circinans*	CBS 675.92	GCA_010015675	NA	NA
*Clathrospora elynae*	CBS 161.51	GCA_010015635	*Carex curvula*	NA
*Corynespora cassiicola*	Phil v1.0	GCA_003016335	NA	Philippines
*Cucurbitaria berberidis*	CBS 394.84	GCA_010015615	NA	NA
*Didymella exigua*	CBS 183.55	GCA_010094145	*Rumex arifolius*	France
*Didymella heteroderae*	28M-1	GCA_011058895	*Homo sapiens*	Brazil
*Fenestella fenestrata*	ATCC 66461	NA *	*Acer* sp.	USA
*Karstenula rhodostoma*	CBS 690.94	GCA_010093485	NA	NA
*Lentithecium fluviatile*	CBS 122367	GCA_010405425	*Populus* sp.	France
*Leptosphaeria maculans*	JN3	GCA_000230375	NA	NA
*Massarina eburnea*	CBS 473.64	GCA_010093635	NA	NA
***Parafenestella ontariensis***	**EI-6**	GCA_021596325 **	***Acer negundo***	**Canada**
*Plenodomus tracheiphilus*	IPT5	GCA_010093695	NA	NA
*Setospaheria turcica*	Et28A	GCA_000359705	NA	NA
*Stagonospora nodorum*	SN15	GCA_000146915	NA	NA

Notes: * proteome was accessed in MycoCosm portal (https://mycocosm.jgi.doe.gov/Fenfe1/Fenfe1.home.html); accessed on 23 March 2022; ** genome annotation is available in MycoCosm portal (https://mycocosm.jgi.doe.gov/Paront1/Paront1.home.html accessed on 25 March 2022).

**Table 3:** Assembly statistics of *P. ontariensis* and other close *Cucurbitariaceae* species.

Features	*C. berberidis* CBS 394.84	*F. fenestrata* ATCC 66461	*P. ontariensis* EI-6
Sequence coverage (×)	145.7	81.9	267.4
Assembly size (MB)	32.9	32.7	34.4
Scaffolds (>1000 bp)	42	22	162
Scaffold N50 (Kb)	6 234	2 290	906
GC content (%)	49.3	51.53	50.79
N of gene models	12 429	11 804	11 748
Repeat rate (%)	2.24	3.91	1.59
BUSCO estimates (%)	97.2	98.1	97.3

## Data Availability

The datasets generated for this study can be found in the GenBank (NCBI), TreeBASE, MycoBank and MycoCosm.

## References

[1] Jaklitsch W., Checa J., Blanco M., Olariaga I., Tello S., Voglmayr H. (2018). A Preliminary Account of the *Cucurbitariaceae*. Stud. Mycol..

[2] Hyde K.D., Jones E.B.G., Liu J.-K., Ariyawansa H., Boehm E.W.A., Boonmee S., Braun U., Chomnunti P., Crous P.W., Dai D.-Q. (2013). Families of Dothideomycetes. Fungal Divers..

[3] Crous P.W., Wingfield M.J., Lombard L., Roets F., Swart W.J., Alvarado P., Carnegie A.J., Moreno G., Luangsa-Ard J., Thangavel R. (2019). Fungal Planet Description Sheets: 951–1041. Persoonia.

[4] Jaklitsch W.M., Voglmayr H. (2020). Fenestelloid Clades of the *Cucurbitariaceae*. Persoonia.

[5] Haridas S., Albert R., Binder M., Bloem J., LaButti K., Salamov A., Andreopoulos B., Baker S., Barry K., Bills G. (2020). 101 Dothideomycetes Genomes: A Test Case for Predicting Lifestyles and Emergence of Pathogens. Stud. Mycol..

[6] Dal’Sasso T.C.D.S., Rody H.V.S., Grijalba P.E., Oliveira L.O. (2021). Genome Sequences and In Silico Effector Mining of *Corynespora cassiicola* CC_29 and *Corynespora olivacea* CBS 114450. Arch. Microbiol..

[7] Rodrigues C.M., Takita M.A., Silva N.V., Ribeiro-Alves M., Machado M.A. (2019). Comparative Genome Analysis of *Phyllosticta citricarpa* and *Phyllosticta capitalensis*, Two Fungi Species that Share the Same Host. BMC Genom..

[8] Stauber L., Prospero S., Croll D. (2020). Comparative Genomics Analyses of Lifestyle Transitions at the Origin of an Invasive Fungal Pathogen in the Genus *Cryphonectria*. Ecol. Evol. Sci..

[9] Lind A.L., Wisecaver J.H., Lameiras C., Wiemann P., Palmer J.M., Keller N.P., Rodrigues F., Goldman G.H., Rokas A. (2017). Drivers of Genetic Diversity in Secondary Metabolic Gene Clusters within a Fungal Species. PLoS Biol..

[10] Amselem J., Cuomo C.A., van Kan J.A., Viaud M., Benito E.P., Couloux A., Coutinho P.M., de Vries R.P., Dyer P.S., Fillinger S. (2011). Genomic Analysis of the Necrotrophic Fungal Pathogens *Sclerotinia sclerotiorum* and *Botrytis cinerea*. PLoS Genet..

[11] Wang B., Liang X., Gleason M.L., Zhang R., Sun G. (2018). Comparative Genomics of *Botryosphaeria dothidea* and *B. kuwatsukai*, Causal Agents of Apple Ring Rot, Reveals both Species Expansion of Pathogenicity–Related Genes and Variations in Virulence Gene Content during Speciation. IMA Fungus.

[12] Lombard V., Golaconda R.H., Drula E., Coutinho P.M., Henrissat B. (2014). The Carbohydrate–Active Enzymes Database (CAZy) in 2013. Nucleic Acids Res..

[13] Zhao Z., Liu H., Wang C., Xu R. (2013). Comparative Analysis of Fungal Genomes Reveals Different Plant Cell Wall Degrading Capacity in Fungi. BMC Genom..

[14] Aime M.C., Miller A.N., Aoki T., Bensch K., Cai L., Crous P.W., Hawksworth D.L., Hyde K.D., Kirk P.M., Lücking R. (2021). How to Publish a New Fungal Species, or Name, Version 3.0. IMA Fungus.

[15] Phookamsak R., Norphanphoun C., Tanaka K., Dai D.-Q., Luo Z.-L., Liu J.-K., Su H.-Y., Bhat D.J., Bahkali A.H., Mortimer P.E. (2015). Towards a Natural Classification of Astrosphaeriella-like Species; Introducing *Astrosphaeriellaceae* and *Pseudoastrosphaeriellaceae* fam. nov. and *Astrosphaeriellopsis*, gen. nov. Fungal Divers..

[16] White T.J., Bruns T.D., Lee S., Taylor J.W., Innis M.A., Gelfand D.H., Sninsky J., White T.J. (1990). Amplification and Direct Sequencing of Fungal Ribosomal RNA Genes for Phylogenetics. PCR Protocols: A Guide to Methods and Applications.

[17] Vilgalys R., Hester M. (1990). Rapid Genetic Identification and Mapping of Enzymatically Amplified Ribosomal DNA from Several *Cryptococcus* Species. J. Bacteriol..

[18] Thompson J.D., Higgins D.G., Gibson T.J. (1994). CLUSTAL W: Improving the Sensitivity of Progressive Multiple Sequence Alignment through Sequence Weighting, Position–Specific Gap Penalties and Weight Matrix Choice. Nucleic Acids Res..

[19] Tamura K., Stecher G., Peterson D., Filipski A., Kumar S. (2013). MEGA6: Molecular Evolutionary Genetics Analysis Version 6.0. Mol. Biol. Evol..

[20] Stamatakis S. (2014). RAxML Version 8: A Tool for Phylogenetic Analysis and Post-Analysis of Large Phylogenies. Bioinformatics.

[21] Huelsenbeck J.P., Ronquist F. (2001). MRBAYES: Bayesian Inference of Phylogenetic Trees. Bioinformatics.

[22] Posada D., Buckley T. (2004). Model Selection and Model Averaging in Phylogenetics: Advantages of Akaike Information Criterion and Bayesian Approaches Over Likelihood Ratio Tests. Syst. Biol..

[23] Darriba D., Posada D., Kozlov A.M., Stamatakis A., Morel B., Flouri T. (2020). ModelTest–NG: A New and Scalable Tool for the Selection of DNA and Protein Evolutionary Models. Mol. Biol. Evol..

[24] Hillis D.M., Bull J.J. (1993). An Empirical Test of Bootstrapping as a Method for Assessing Confidence in Phylogenetic Analysis. Syst. Biol..

[25] Rambaut A. (2018). FigTree.

[26] Bolger A.M., Lohse M., Usadel B. (2014). Trimmomatic: A Flexible Trimmer for Illumina Sequence Data. Bioinformatics.

[27] Bankevich A., Nurk S., Antipov D., Gurevich A.A., Dvorkin M., Kulikov A.S., Lesin V.M., Nikolenko S.I., Pham S., Prjibelski A.D. (2012). SPAdes: A New Genome Assembly Algorithm and its Applications to Single–Cell Sequencing. J. Comput. Biol..

[28] Mikheenko A., Prjibelski A., Saveliev V., Antipov D., Gurevich A. (2018). Versatile Genome Assembly Evaluation with QUAST–LG. Bioinformatics.

[29] Smit A.F.A., Hubley R., Green P. 2013–2015 RepeatMasker Open–4.0. http://www.repeatmasker.org.

[30] Bao W., Kojima K.K., Kohany O. (2015). Repbase Update, a Database of Repetitive Elements in Eukaryotic Genomes. Mob. DNA.

[31] Simão F.A., Waterhouse R.M., Ioannidis P., Kriventseva E.V., Zdobnov E.M. (2015). BUSCO: Assessing Genome Assembly and Annotation Completeness with Single–Copy Orthologs. Bioinformatics.

[32] Stanke M., Morgenstern B. (2005). AUGUSTUS: A Web Server for Gene Prediction in Eukaryotes that Allows User–Defined Constraints. Nucleic Acids Res..

[33] Cantarel B.L., Korf I., Robb S.M.C., Parra G., Ross E., Moore B., Holt C., Alvarado A.S., Yandell M. (2008). MAKER: An Easy-to-Use Annotation Pipeline Designed for Emerging Model Organism Genomes. Genome Res..

[34] Huerta-Cepas J., Szklarczyk D., Heller D., Hernández-Plaza A., Forslund S.K., Cook H.V., Mende D.R., Letunic I., Rattei T., Jensen L.J. (2019). EggNOG 5.0: A Hierarchical, Functionally and Phylogenetically Annotated Orthology Resource Based on 5090 Organisms and 2502 Viruses. Nucleic Acids Res..

[35] Yin Y., Mao X., Yang J., Chen X., Mao F., Xu Y. (2012). dbCAN: A Web Resource for Automated Carbohydrate-Active Enzyme Annotation. Nucleic Acids Res..

[36] Wolf T., Shelest V., Nath N., Shelest E. (2016). CASSIS and SMIPS: Promoter–Based Prediction of Secondary Metabolite Gene Clusters in Eukaryotic Genomes. Bioinformatics.

[37] Grigoriev I.V., Nikitin R., Haridas S., Kuo A., Ohm R.A., Otillar R., Riley R., Salamov A.A., Zhao X., Korzeniewski F. (2014). MycoCosm Portal: Gearing up for 1000 Fungal Genomes. Nucleic Acids Res..

[38] Emms D.M., Kelly S. (2015). OrthoFinder: Solving Fundamental Biases in Whole Genome Comparisons Dramatically Improves Orthogroup Inference Accuracy. Genome Biol..

[39] Katoh K., Standley D.M. (2013). MAFFT Multiple Sequence Alignment Software Version 7: Improvements in Performance and Usability. Mol. Biol. Evol..

[40] Price M.N., Dehal P.S., Arkin A.P. (2010). FastTree 2-Approximately Maximum–Likelihood Trees for Large Alignments. PLoS ONE..

[41] Jain C., Koren S., Dilthey A.T., Phillippy A.M., Aluru S. (2018). A Fast Adaptive Algorithm for Computing Whole–Genome Homology Maps. Bioinformatics.

[42] Cabanettes F., Klopp C.D. (2018). GENIES: Dot Plot Large Genomes in an Interactive, Efficient and Simple Way. PeerJ.

[43] Wanasinghe D.N., Phookamsak R., Jeewon R., Li W.J., Hyde K.D., Gareth Jones E.B., Erio C., Promputtha I. (2017). A Family Level rDNA Based Phylogeny of *Cucurbitariaceae* and *Fenestellaceae* with Descriptions of New *Fenestella* Species and *Neocucurbitaria* gen. nov. Mycosphere.

[44] Mohanta T.K., Bae H. (2015). The Diversity of Fungal Genome. Biol. Proced. Online.

[45] Rao S., Nandineni M.R. (2017). Genome Sequencing and Comparative Genomics Reveal a Repertoire of Putative Pathogenicity Genes in Chilli Anthracnose Fungus *Colletotrichum truncatum*. PLoS ONE.

[46] Li S., Song Q., Ji P., Cregan P. (2015). Draft Genome Sequence of *Phomopsis longicolla* Type Strain TWH P74, a Fungus Causing Phomopsis Seed Decay in Soybean. Genome Announc..

[47] Hane J.K., Williams A.H., Oliver R.P., Pöggeler S., Wöstemeyer J. (2011). 9 Genomic and Comparative Analysis of the Class Dothideomycetes. Evolution of Fungi and Fungal–Like Organisms. The Mycota, 14.

[48] Khaldi N., Collemare J., Lebrun M.H., Wolfe K.H. (2008). Evidence for Horizontal Transfer of a Secondary Metabolite Gene Cluster Between Fungi. Genome Biol..

[49] Kadeawi S., Nasution A., Hairmansis A., Telebanco-Yanoria M.J., Obara M., Hayashi N., Fukuta Y. (2021). Pathogenicity of Isolates of the Rice Blast Pathogen (*Pyricularia oryzae*) from Indonesia. Plant Dis..

[50] Kamel S., Cherif M., Hafez M., Despins T., Aboukhaddour R. (2019). *Pyrenophora tritici–repentis* in Tunisia: Race Structure and Effector Genes. Front. Plant Sci..

[51] Oide S., Turgeon B.G. (2020). Natural Roles of Nonribosomal Peptide Metabolites in Fungi. Mycoscience.

[52] Wang X., Wei J., Huang L., Kang Z. (2011). Re-evaluation of Pathogens Causing Valsa Canker on Apple in China. Mycologia.

[53] Ohm R.A., Feau N., Henrissat B., Schoch C.L., Horwitz B.A., Barry K.W., Condon B.J., Copeland A.C., Dhillon B., Glaser F. (2012). Diverse Lifestyles and Strategies of Plant Pathogenesis Encoded in the Genomes of Eighteen Dothideomycetes fungi. PLoS Pathog..

[54] Zhou J., Li X., Huang P.W., Dai C.C. (2017). Endophytism or Saprophytism: Decoding the Lifestyle Transition of the Generalist Fungus *Phomopsis liquidambari*. Microbiol. Res..

[55] Yang Y., Liu X., Cai J., Chen Y. (2019). Genomic Characteristics and Comparative Genomics Analysis of the Endophytic Fungus *Sarocladium brachiariae*. BMC Genom..

